# Effect of a Video-Guided Swallowing Exercise Program on Adherence in Stroke Patients with Dysphagia: A Randomized Controlled Trial

**DOI:** 10.1007/s00455-025-10907-2

**Published:** 2025-12-01

**Authors:** Jumpon Puangdech, Poungkaew Thitisakulchai, Vilai Kuptniratsaikul, Warinda Onkampa

**Affiliations:** 1 Department of Rehabilitation Medicine, Faculty of Medicine Siriraj Hospital, Mahidol University, 2 Wanglang Road, Bangkoknoi, Bangkok, 10700 Thailand; 2 Present Address: Bangsaphan Hospital, Prachuap Khiri Khan, Thailand

**Keywords:** Stroke, Dysphagia, Swallowing, Treatment adherence and compliance, Multimedia

## Abstract

To compare swallowing exercise adherence, swallowing function, and media satisfaction between stroke patients with dysphagia using a video-guided exercise program on mobile electronic devices versus those using a handbook. Randomized controlled trial. Forty-four stroke patients with dysphagia were randomized to either a video-guided intervention group (*n* = 23) or a handbook-based control group (*n* = 21). The prescribed exercises included lip exercises, tongue range of motion and strengthening, chin tuck against resistance (CTAR), and Masako exercises, performed as three sets of ten repetitions daily. Baseline swallowing function was assessed using the Functional Oral Intake Scale (FOIS), and adherence was self-reported via logbooks. Outcomes were reassessed at 1-month follow-up. Forty participants completed the study (21 intervention, 19 control). Baseline characteristics were comparable, except for a higher prevalence of left hemiparesis and a longer time since stroke onset in the intervention group. Exercise adherence was significantly higher in the intervention group (median 42.9% [IQR 24.6, 78.4]) compared to the control group (19.4% [IQR 0, 38.7]; *p* = 0.009). Both groups showed FOIS score improvements, with no significant between-group difference. Media satisfaction did not differ significantly. A video-guided swallowing exercise program delivered via mobile devices significantly improved adherence compared to handbook-based instruction. This approach may offer a feasible and accessible strategy for post-stroke dysphagia rehabilitation.

## Introduction

Dysphagia is a common and serious complication following stroke, with substantial implications for both health and quality of life. Globally, its prevalence is estimated at approximately 42–47%, with regional rates ranging from 40% to 64% [1, 2]. In Thailand, a study reported that about 15% of patients in the acute phase of stroke experience dysphagia, and 20% of these cases persist beyond one year [3]. Post-stroke dysphagia is associated with more than a fourfold increase in the risk of pneumonia and mortality [2]. It also contributes to reduced quality of life, greater caregiver burden, prolonged hospital stays, and increased healthcare costs [4, 5]. 

Swallowing therapy for stroke patients includes dietary modifications and postural adjustments, which aim to improve swallowing safety and efficiency, as well as swallowing exercises, which are essential for enhancing muscle strength and coordination [6]. Although home-based swallowing exercises play a critical role in post-stroke rehabilitation, adherence remains suboptimal. Surveys suggest that only about 65% of patients partially adhere to prescribed regimens [7], while more objective assessments report adherence rates as low as 28% when ≥ 70% task completion is required [8]. Poor adherence has been associated with higher risks of aspiration, malnutrition, pneumonia, and other unfavorable outcomes [9]. Multiple factors contribute to this challenge, including difficulty remembering the exercises, unclear instructions, low motivation, insufficient caregiver support, and underlying mental health conditions [6]. Addressing these obstacles—through improved patient education, timely reminders, motivational strategies, and greater caregiver involvement—is essential to enhancing rehabilitation outcomes.

During hospitalization, occupational therapists typically prescribe dysphagia exercise programs, which are often supplemented by printed handbooks to support home-based exercise training after discharge. These materials, usually in the form of brochures or booklets, provide essential information about dysphagia and its complications, along with instructions for therapeutic exercises. They describe the purpose, technique, and recommended dosage of each exercise, often accompanied by illustrations to facilitate understanding. The primary function of these handbooks is to reinforce in-hospital teaching, enhance patient self-efficacy, and serve as a practical reference for patients and caregivers. However, exercise adherence based on handbook use remains limited. For example, Kara et al. found no significant difference in adherence between patients receiving printed materials and those given only verbal instruction [10], suggesting that traditional formats may not sufficiently support long-term engagement. These limitations underscore the need for more interactive and accessible strategies to improve continuity in home-based rehabilitation.

Video-based exercise programs offer a promising approach by providing visual and auditory cues that enhance engagement and facilitate learning. Evidence suggests that video-guided instruction improves exercise adherence, confidence, and accuracy compared to paper-based materials. In stroke survivors, a mobile video–guided home exercise program achieved a 75.6% adherence rate and higher self-efficacy, significantly outperforming the 55.2% adherence observed in those using printed instructions [11]. Similar benefits have been reported in upper limb rehabilitation, where video guidance led to higher exercise completion rates and improved patient confidence in performing movements correctly [12]. Additionally, a systematic review in dysphagic head and neck cancer survivors found that home-based telerehabilitation programs improved adherence, swallowing safety, and swallowing outcomes, while also being well-received and cost-effective [13]. 

Despite these encouraging findings, the effectiveness of video-based interventions specifically for improving adherence to post-stroke swallowing exercises remains uncertain. Although prior studies have compared video- and paper-based programs across various rehabilitation domains [11, 14, 15], randomized controlled trials targeting stroke patients with dysphagia are lacking.

This study aims to evaluate the effectiveness of a video-guided swallowing exercise program in enhancing adherence among stroke patients with dysphagia. The primary objective is to compare adherence rates between patients using video-based training via electronic devices and those following printed handbook instructions. Secondary objectives include evaluating swallowing function and patient satisfaction with the respective training methods.

## Materials and methods

### Study Design

A randomized, controlled, assessor-blinded clinical trial was conducted at a tertiary care hospital in Thailand from January 2021 to December 2023. The study was approved by the institutional review board (COA 870/2020) and registered in the Thai Clinical Trial Registry (TCTR20201207003).

### Selection Criteria

Stroke patients with dysphagia were enrolled based on the following inclusion criteria: failure of the 90 mL water swallowing test, which has been recommended as a screening tool for aspiration in stroke patients [16]; stroke onset within six months; age over 18 years; medical stability; ability to perform swallowing exercises, determined by cognitive and visual capacity to understand and follow instructions; and access to a mobile electronic device such as a smartphone or tablet. Participants who failed the water swallowing test subsequently underwent a clinical swallowing assessment by a rehabilitation physician not involved in the study to confirm the presence of swallowing dysfunction. However, due to financial constraints, instrumental evaluations such as videofluoroscopic or endoscopic swallowing studies were not performed. Patients were excluded if they had visual impairments, dysphagia unrelated to stroke, or required transfer to another healthcare facility. Participants unable to continue the program for medical reasons or those who requested withdrawal were discontinued from the study.

### Sample Size

Based on the study by Emmerson et al.[15], a sample size of 44 participants was determined to achieve 80% power with a 95% confidence interval, accounting for a 10% dropout rate.

### Procedure

Stroke patients with dysphagia who met the inclusion criteria were invited to participate in the study. All participants received a detailed explanation of the procedures and provided informed consent prior to enrollment. They were then randomly assigned to either the intervention or control group using a computer-generated randomization process, and baseline characteristics were recorded. Although lesion location and size are recognized as important factors influencing the severity and recovery of post-stroke dysphagia [17–19, 20], imaging data were not collected as part of the study due to resource constraints commonly encountered in clinical settings in Thailand.

### Control Group

Participants in the control group received a printed handbook illustrating five standard swallowing exercises: lip exercises, tongue range of motion and strengthening, chin tuck against resistance (CTAR), and the Masako maneuver.

Lip and tongue exercises were prescribed to improve oral musculature strength and mobility, essential for bolus control and transit during the oral phase of swallowing. Lip exercises included six targeted movements: wide mouth opening, broad smiling to expose the teeth, lip pursing (as if whistling), firm lip closure, sucking the cheeks inward, and puffing out the cheeks. Tongue range of motion tasks involved protrusion, circular movement around the lips, and elevation toward the palate followed by sweeping toward the uvula. Strengthening tasks consisted of pressing the tongue forcefully against the inner cheeks bilaterally; resistance was increased by applying external finger pressure as participants progressed. Each exercise was performed for ten repetitions per session, with one full set comprising all prescribed movements. These exercises are commonly used in dysphagia rehabilitation to enhance lip seal, intraoral pressure, and bolus manipulation, and have demonstrated efficacy in improving oral-phase swallowing function, particularly in post-stroke populations [21]. 

The CTAR exercise was performed using resistance from an inflatable ball, following established protocols. When unavailable, alternatives such as a rolled towel or the participant’s fist were used. The exercise included both isometric (chin pressed against resistance and held for 30 s) and isokinetic (chin pressed and released for 30 repetitions) components, which together comprised one set. For participants unable to complete 30 repetitions, a modified set of 10 was permitted. This exercise targets the suprahyoid muscles responsible for hyolaryngeal elevation and has shown comparable or superior muscle activation to the Shaker exercise [22]. 

The Masako maneuver, or tongue-hold swallow, was prescribed to enhance pharyngeal strength and coordination. Participants were instructed to gently protrude the tongue and hold it in place with their teeth or lips while performing a dry swallow. For those unable to maintain tongue position using lip strength alone, manual support with the hand was recommended. This exercise was performed for ten repetitions per set. Repetitive practice of this maneuver has been shown to improve swallowing function in stroke patients with dysphagia [23]. 

All exercises, except for CTAR, were prescribed at ten repetitions per set, three sets per day, for one month. This dosage aligns with commonly reported clinical practice. A systematic review of dysphagia rehabilitation protocols indicated that oromotor and tongue exercises are frequently prescribed in sets of 10 repetitions. In contrast, CTAR is typically administered as three isometric holds and 30 isokinetic repetitions per session, performed three to seven times per week [24], supporting the use of a similar dosing framework.

### Intervention Group

Participants in the intervention group accessed the same set of exercises through a video-based program delivered via mobile electronic devices. The video featured a simulated patient performing each exercise in real time, allowing participants to follow along. A voiceover narration guided them throughout the session—providing instructions, signaling when to start and rest, counting repetitions, and reminding them to complete the exercises three times daily. Countdown intervals were included to mark 30-second rest periods between movements within the same category, promoting structured and consistent practice. Exercises in different categories were delivered in separate videos, allowing participants to choose when to perform each one throughout the day.

### Exercise Monitoring and Follow-up

Prior to discharge, all participants were assessed to ensure accurate performance of the prescribed exercises. They were instructed to refrain from using additional training media or seeking dysphagia-related treatment at other healthcare facilities during the study period. Each participant received a self-reported logbook to document their adherence to the home exercise regimen. To support compliance, weekly reminder phone calls were made to participants in both groups, encouraging them to maintain their logbooks and continue the prescribed exercises as instructed.

At the one-month follow-up, a blinded assessor evaluated each participant to assess exercise adherence (based on logbook entries), swallowing function, and satisfaction with the assigned instructional media.

### Outcome Measurements

#### Swallowing Exercise Adherence

Given the absence of a standardized metric for measuring adherence to swallowing exercises [25, 26], a self-reported logbook was employed to monitor adherence throughout the study. This method has been shown to provide greater accuracy than retrospective surveys or interviews [27] by allowing real-time or near-real-time documentation.

Participants in both groups were instructed to record their performance for each prescribed exercise during every session. For each exercise category, adherence was categorized into three levels: no repetitions performed; less than 50% of the prescribed repetitions completed; or at least 50% of the prescribed repetitions completed.

Exercise adherence was then quantified as the percentage of days in which participants met the exercise target—defined as completing ≥ 50% of the recommended repetitions for all prescribed exercises across three sets—relative to the total number of assigned days.

1 $$\begin{aligned} & Swallowing\;exercise\;adherence\;(percentage\;of\;days\;meeting\;the\;exercise\;target) \\ & {\text{ = }}\frac{{{\mathrm{Number}}\;{\mathrm{of}}\;{\mathrm{days}}\;{\mathrm{meeting}}\;{\mathrm{the}}\;{\mathrm{exercise}}\;{\mathrm{target}}}}{{{\mathrm{Total}}\;{\mathrm{assigned}}\;{\mathrm{days}}}} \times {\mathrm{100}} \\ \end{aligned}$$

#### Swallowing Function

Swallowing function was assessed using the Functional Oral Intake Scale (FOIS) by a blinded occupational therapist to minimize bias. FOIS is a reliable and clinically meaningful tool for evaluating swallowing function in individuals with neurological impairments, particularly those recovering from stroke. It has demonstrated excellent interrater reliability and sensitivity to changes in oral intake over time. FOIS has also been validated against other established assessments, such as the Mann Assessment of Swallowing Ability (MASA), showing strong criterion validity and supporting its use as a functional outcome measure [28]. A systematic review has further identified FOIS as one of the most commonly used tools for monitoring dysphagia recovery in stroke populations [17]. The scale consists of seven levels, ranging from level 1 (nothing by mouth) to level 7 (total oral diet with no restrictions), and can be dichotomized to distinguish between tube-dependent patients (FOIS ≤ 3) and those with full oral intake (FOIS >3).

#### Media Satisfaction

Media satisfaction was evaluated using a 0–10 Numeric Rating Scale, where 0 indicated no satisfaction and 10 represented the highest level of satisfaction.

### Statistical Analysis

Data were analyzed using PASW Statistics for Windows, version 18.0 (SPSS Inc, Chicago, IL, USA). Quantitative data were summarized as mean ± standard deviation or median with interquartile range, depending on distribution, while categorical data were reported as frequencies and percentages. Continuous variables were analyzed using independent t-tests or Mann–Whitney U tests, as appropriate. FOIS scores and other discrete numeric data were assessed using the Mann–Whitney U or Wilcoxon signed-rank test. Categorical variables were compared using Fisher’s exact test, Pearson’s chi-square test, or McNemar’s test, as applicable. No corrections for multiple comparisons (e.g., Bonferroni) were applied, as the analyses did not involve comparisons across multiple groups or time points. Statistical significance was set at *p* < 0.05.

## Results

**Fig. 1:**
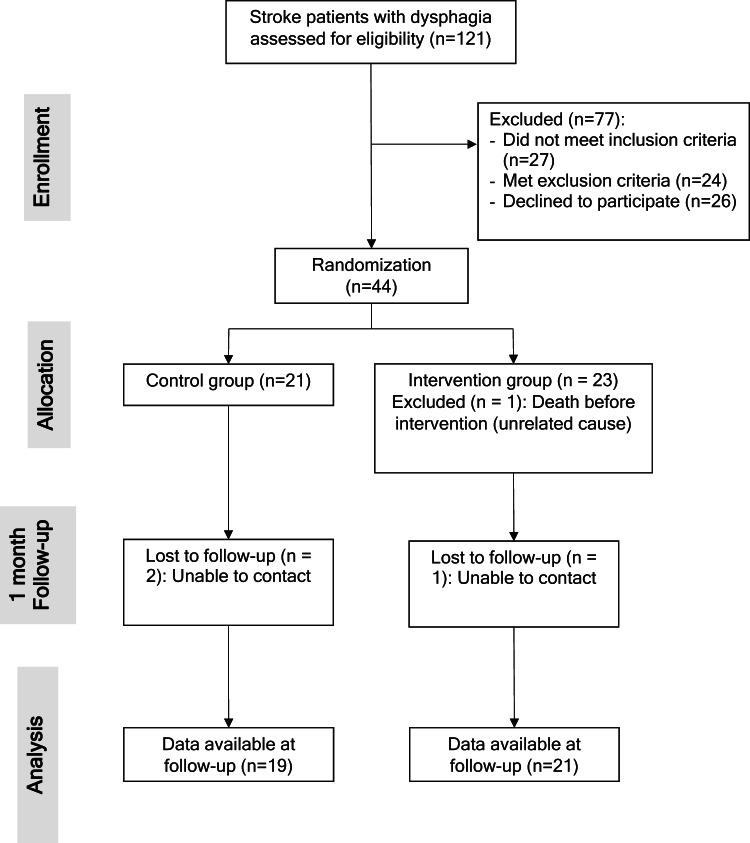
Participants flow through the trial

A total of 44 stroke patients with dysphagia were enrolled and randomly assigned to either the intervention or control group. One participant in the intervention group passed away from an unrelated medical condition before initiating the program and was therefore excluded from the study, leaving 43 participants for analysis. At the one-month follow-up, three additional participants were lost to follow-up—one from the intervention group and two from the control group. Consequently, data from 21 participants in the intervention group and 19 in the control group were available at follow-up (Fig. 1). Following an intention-to-treat approach, analyses were conducted including all 43 participants, with missing adherence data replaced with 0 and missing FOIS scores carried forward from baseline values.

Baseline characteristics were comparable between the intervention and control groups, with no significant differences in age, gender, body mass index (BMI), Thai Mental State Examination (TMSE) scores, education level, caregiver support, or stroke type (predominantly first-ever ischemic strokes). Initial FOIS scores and stroke severity at admission, as measured by the NIH Stroke Scale (NIHSS), both recognized predictors of dysphagia recovery, [17–19] were also similar between groups. There was no statistically significant difference in the proportion of participants with NIHSS scores below or above 12, a commonly used threshold for predicting persistent post-stroke dysphagia [29]. However, the intervention group had a significantly higher prevalence of left-sided hemiparesis (suggesting right-hemispheric stroke) and a longer time since stroke onset (*p* = 0.039 and *p* = 0.014, respectively). Patterns of mobile device usage, including daily screen time and specific features accessed, did not differ significantly between groups (Table 1).

At the one-month follow-up, swallowing exercise adherence was evaluated using self-reported logbooks (Table 2). The intention-to-treat analysis showed a significantly higher median adherence rate in the intervention group (42.9%) compared to the control group (19.4%) (*p* = 0.009). A per-protocol analysis yielded consistent results (*p* = 0.010; data not shown), supporting the consistency of the results.

Baseline swallowing function, assessed using FOIS, showed no significant differences between groups. After one month, both groups demonstrated significant within-group improvement; however, no significant differences were observed between groups (*p* = 1.000) (Table 3). The number of participants achieving full oral intake increased in both groups, with no significant between-group difference (*p* = 0.835) (Table 4).

The media satisfaction score, assessed using a numeric rating scale, indicated high satisfaction in both groups. The intervention group had a mean score of 8.7 ± 1.4, while the control group had a mean score of 8.7 ± 1.3, with no significant difference between groups (*p* = 0.958; details not tabulated).

Adverse events included fatigue (three cases in the intervention group and six in the control group) and pain (one case in the control group). The overall incidence of adverse events was comparable between groups (*p* = 0.148; details not tabulated).

## Discussion

The primary results indicate that patients who participated in a video-guided, home-based swallowing exercise program using a mobile electronic device after discharge demonstrated better adherence compared to those who received a handbook-based program. This finding is consistent with previous research, including the study by Lambert et al. on individuals with musculoskeletal conditions and the pilot study by Chung et al. on physiotherapy for stroke patients [11, 14]. Compared with video-based programs, paper handouts may present practical limitations such as misplacement, reduced engagement, and challenges in interpreting static images without accompanying motion or audio. Although the handbooks used in this study included photographs and written descriptions of each exercise, qualitative interviews with stroke survivors in previous studies have shown that static images may be insufficient for fully conveying the dynamic aspects of movement. This can lead to uncertainty in execution and reduced motivation to perform exercises correctly. In contrast, video-guided programs offer continuous visual and auditory demonstrations that more effectively illustrate movement quality and sequencing, enhance understanding, and improve convenience through on-demand access [30]. 

The median swallowing exercise adherence rate in the intervention group was 42.9%, which is notably lower than the adherence rates reported in previous studies—78% for an app with remote support in individuals with musculoskeletal conditions, [14] and 73.7% for mobile video-guided home exercise programs in stroke patients [11]. This discrepancy may partly reflect differences in adherence assessment methods. These prior studies relied on interview-based self-reports, which are prone to overestimation due to recall and reporting bias [31]. In contrast, the present study employed self-reported logbooks, which, although still subject to some degree of response bias, have shown higher concordance with objective measures—such as electronic monitoring—compared to interviews. Garber et al. reported that diary-based self-report methods were moderately to highly concordant with electronic adherence measures in 75% of comparisons, while interview-based methods showed no high concordance in similar comparisons [27]. Although not equivalent to electronic monitoring, logbooks may offer a more accurate reflection of actual adherence behavior than interviews, particularly in unsupervised home-based settings.

Although the intervention group demonstrated significantly higher adherence compared to the control group, no significant between-group differences in swallowing function, as measured by the FOIS, were observed after one month. Notably, both groups showed significant within-group improvements in FOIS scores at follow-up. Given that participants were enrolled during the acute to early subacute phase post-stroke, this improvement may be partly attributed to spontaneous recovery of swallowing function, as previously reported in longitudinal studies of post-stroke dysphagia, including the study by Arreola et al., which documented substantial natural improvement over the first three months following stroke [32]. Additionally, baseline differences may have influenced the outcomes. The intervention group had a higher proportion of left-sided hemiparesis, suggesting right-hemispheric lesions, which have been associated with more severe pharyngeal phase impairments, including delayed swallow initiation and reduced airway protection [33, 34]. Although the median time from stroke onset to enrollment was longer in the intervention group (16 days) compared to the control group (8 days), both timeframes fell within the early recovery period, potentially limiting the extent of differential effects on swallowing outcomes.

The limited sensitivity of the FOIS in detecting subtle physiological improvements may have also influenced the findings. Although FOIS is a reliable and clinically meaningful tool for assessing functional oral intake, it primarily reflects dietary status and may overlook nuanced improvements in swallowing physiology, such as enhanced muscular strength or coordination [35]. Alternative outcome measures—such as objective assessments of muscle performance or time to transition from tube feeding to full oral intake—may offer greater sensitivity in detecting changes resulting from therapeutic interventions.

Another contributing factor to the overall low adherence and the non-significant difference in swallowing function between groups may have been the limited integration of fundamental exercise science principles—namely, overload, progression, specificity, individualization, and feedback—within the prescribed exercise protocol. All participants were assigned the same fixed regimen without adjustments in intensity, progression based on performance, or mechanisms for providing feedback. This lack of personalization and progression may have reduced participant engagement and confidence, while the static nature of the exercises may have been insufficient to elicit meaningful physiological adaptations or functional gains.

In contrast, rehabilitation programs that incorporate these principles have demonstrated greater effectiveness. The McNeill Dysphagia Therapy Program (MDTP), for example, applies a task-specific, progressively challenging approach to swallowing rehabilitation. In a randomized controlled trial, Carnaby et al. reported that patients undergoing MDTP achieved significantly greater improvements in FOIS scores and faster return to pre-stroke dietary levels compared to those receiving standard or sham interventions [36]. Similarly, interventions using individualized digital coaching, mobile applications with sEMG biofeedback, and progressive resistance devices have shown higher adherence rates and more robust gains in swallowing function [37–40]. 

These findings underscore the importance of integrating exercise science principles into the design of dysphagia rehabilitation protocols. Future studies should consider tailoring exercises to individual capabilities, incorporating progressive overload based on patient performance, ensuring task specificity, and embedding feedback mechanisms—whether through clinician oversight, digital platforms, or biofeedback technologies. Such enhancements may not only improve adherence but also optimize the therapeutic efficacy of home-based swallowing interventions.

However, the implementation of these more elaborate, resource-intensive protocols can be challenging in settings with limited staffing, equipment, or funding. Many facilities—particularly in Thailand—lack the personnel or technological infrastructure required to deliver truly individualized, feedback-driven programs. These limitations represent a significant barrier to the widespread adoption of such regimens and must be addressed to enable scalable, effective interventions.

Regarding patient satisfaction, the value was high in both groups, with no significant differences observed. This may reflect the provision of clear and accessible instructional materials in each format. The well-designed handbooks likely supported satisfaction among older adults less familiar with mobile devices, while the video-based program was appreciated for its clarity and guided instruction. However, some participants reported needing caregiver assistance to access the videos. In contrast, handbooks were easier to use independently but were perceived as less engaging. These findings suggest that preferences for instructional delivery vary depending on factors such as technological familiarity, cognitive capacity, and the level of caregiver support. Offering multiple formats may therefore improve adherence by allowing patients to engage with the modality that best aligns with their individual needs and circumstances.

### Strengths and Limitations

This randomized trial is the first to examine the impact of instructional media on adherence to swallowing exercises in stroke patients with dysphagia. The findings suggest that video-based programs delivered via mobile devices significantly enhance adherence, highlighting their potential role in tele-rehabilitation.

However, no significant differences in swallowing function were observed between groups. This may be attributed to the limited sensitivity of the FOIS scale in detecting subtle physiological changes and the lack of integration of fundamental exercise science principles into the prescribed regimen. These limitations may have reduced both participant engagement and the potential for functional gains. Future studies should consider using more sensitive outcome measures alongside tailored, progressive exercise protocols that incorporate feedback mechanisms to better support recovery.

Additional limitations include the short follow-up period, which precludes evaluation of long-term or carryover effects, and the cognitive and technological demands of the intervention, which may limit its generalizability to individuals with cognitive deficits or minimal caregiver support. Addressing these challenges may require the development of more adaptable delivery strategies, such as simplified digital interfaces, caregiver-facilitated models, or hybrid systems that incorporate personalization, reminders, and motivational supports to enhance adherence and therapeutic outcomes.

## Conclusion

This study found that stroke patients with dysphagia who engaged in a video-based swallowing exercise program via mobile electronic devices demonstrated significantly greater adherence compared to those using handbook-based instructions. However, no significant differences were observed between groups in swallowing function or media satisfaction. These findings suggest that mobile video-guided programs may offer a feasible and engaging approach to support home-based rehabilitation and improve patients’ quality of life.

**Table 1:** Baseline characteristics compared between the intervention and control groups

Variables	Control group (*N* = 21)	Intervention group (*N* = 22)	*p*-value
Age (years), mean ± SD	69.0 ± 12.2	69.9 ± 10.1	0.791^a^
Male, n (%)	14(66.7)	13(59.1)	0.607^b^
BMI (kg/m^2^), mean ± SD	23.8 ± 3.3	24.1 ± 4.4	0.815^a^
TMSE, mean ± SD	23.5 ± 4.3	23.9 ± 3.4	0.774^a^
*Education, n *(%)			0.119^b^
≤ 12 years	17 (81.0)	13 (59.1)	
> 12 years	4 (19.0)	9 (40.9)	
*Caregiver, n *(%)			0.686^b^
Spouse	7 (33.3)	10 (45.5)	
Child and sibling	11 (52.4)	10 (45.5)	
Other	3 (14.3)	2 (9.1)	
*Stroke-related characteristics*			
Stroke type (Ischemic/Hemorrhagic)	19/2	18/4	0.664^c^
Stroke episode (First/Recurrent)	18/3	19/3	1.000^c^
Side of weakness (Left/Right)	11/10	18/4	**0.039**^**b**^ *
NIHSS at admission, median (IQR)	8 (5,11)	11 (7,15)	0.079^d^
< 12, n (%)	17 (81)	15 (68.2)	0.337^b^
≥ 12, n (%)	4 (19)	7 (31.8)	
Time from stroke onset (days), median (IQR)	8(5,22)	16(9.5,37)	**0.014**^**d**^ *
Mobile device usage time, n (%)			0.396^b^
≤ 1 h./day	15 (71.4)	13 (59.1)	
> 1 h./day	6 (28.6)	9 (40.9)	
Mobile device feature use, n (%)			0.850^b^
Calls only	8 (38.1)	9 (40.9)	
Use applications beyond calling	13 (61.9)	13 (59.1)	

^a^Independent t-test, ^b^Pearson Chi-square test, ^c^Fisher’s Exact test, ^d^Mann-Whitney U test

*Indicates statistical significance (*p*-value < 0.05)

Of the 44 randomized participants, one in the intervention group died from an unrelated medical condition before starting the program and was excluded from baseline analysis. Baseline characteristics of the remaining 43 participants are presented using an intention-to-treat approach. Three participants were lost to follow-up and did not complete post-intervention assessments

*BMI* body mass index; *TMSE* Thai Mental State Examination; *NIHSS* National Institutes of Health Stroke Scale; *IQR* interquartile range

**Table 2:** Swallowing exercise adherence after 1-month follow-up

Swallowing exercise adherence, (%)	Control group (*N* = 21)	Intervention group (*N* = 22)	*p*-value
Median (IQR)	19.4 (0, 38.7)	42.9 (24.6, 78.4)	0.009^a^ *
Min., Max.	0, 100	0, 100	

^a^Mann-Whitney U test

*Indicates statistical significance (*p*-value < 0.05)

Includes all participants who entered the intervention phase (*N* = 43) per ITT analysis

*IQR* interquartile range

**Table 3:** Swallowing function at baseline and 1-month follow-up

FOIS, Median (IQR)	Control group	Intervention group	*p*-value
	(*N* = 21)	(*N* = 22)	(between groups)
At baseline	2 (1,3)	2 (2,4)	0.221^a^
At 1 month follow up	6 (2.5,7)	6 (3,7)	1.000^a^
*p*-value (within group)	< 0.001^b^ *	< 0.001^b^ *	

^a^Mann-Whitney U test, ^b^Wilcoxon signed-rank test

^*^Indicates statistical significance (*p*-value < 0.05)

Includes all participants who entered the intervention phase (*N* = 43) per ITT analysis

*FOIS* Functional Oral Intake Scale; *IQR* interquartile range

**Table 4:** Route of feeding at baseline and 1-month follow-up

	Control group (*N* = 21)		Intervention group (*N* = 22)		*p*-value (between groups)
	Tube dependent, *n* (%)	Total oral feeding, *n* (%)	Tube dependent, *n* (%)	Total oral feeding, *n* (%)	
At baseline	17 (81.0)	4 (19.0)	16 (72.7)	6 (27.3)	0.721^a^
At 1 month follow-up	7 (33.3)	14 (66.7)	8 (36.4)	14 (63.6)	0.835^b^
*p*-value (within group)	0.002^c^ *		0.008^c^ *		

^a^Fisher’s Exact test, ^b^Pearson Chi-square test, ^c^McNemar’s test

^*^Indicates statistical significance (*p*-value < 0.05)

Includes all participants who entered the intervention phase (*N* = 43) per ITT analysis

## Data Availability

The datasets generated and/or analyzed during the current study are not publicly available but are available from the corresponding author on reasonable request.
